# Flow injection analysis of nitrogen dioxide using a galvanic detector

**DOI:** 10.1155/S1463924698000030

**Published:** 1998

**Authors:** Shan-jun Liu, Han-xi Shen, Jian-xing Feng, M. Tubino

**Affiliations:** 1 Department of Chemistry Nankai University Tianjin 300071 China; 2 Department of Environmental Sciences Nankai University Tianjin 300071 China; 3 Instituto de Quimica Universidade Estadual de Campinas Caixa Postal 6154 Campinas SP CEP 13083-970 Brazil

## Abstract

A flow injection configuration (FIA) based on a galvanic detector for the determination of nitrogen dioxide is described. The gaseous sample is directly injected into a gaseous carrier. The sample is transported toward the detector. The steady state measurements are not required to obtain the reproducible peak signals. The features of FIA are compared with that of continuous flow monitoring application. The flow injection system is simple, rapid and capable of detecting NO_2_ in the range of 1-500ppm (v/v). The measuring range and sensitivity of the galvanic detector in FIA depend on the sample volume. A relative standard deviation is 2.4% (n = 10) for 200ppm (v/v) of nitrogen dioxide. The sampling frequency is about 24 h^-1^


Journal of Automatic Chemistry, Vol. 20, No. 1 (January–February 1998) pp. 17–21

### Flow injection analysis of nitrogen dioxide using a galvanic detector

**Shan-jun Liu,*'‡ Han-xi Shen,* Jian-xing Feng† and M. Tubino‡**

** Department of Chemistry, † Department of Environmental Sciences, Nankai University, Tianjin, 300071, PR China; ‡ Instituto de Química, Universidade Estadual de Campinas, Caixa Postal 6154, CEP 13083-970, Campinas, SP, Brazil*

*A flow injection configuration (FIA) based on a galvanic detector for the determination of nitrogen dioxide is described. The gaseous sample is directly injected into a gaseous carrier. The sample is transported toward the detector. The steady state measurements are not required to obtain the reproducible peak signals. The features of FIA are compared with that of continuous flow monitoring application. The flow injection system is simple, rapid and capable of detecting $NO_2$ in the range of 1–500 ppm (v/v). The measuring range and sensitivity of the galvanic detector in FIA depend on the sample volume. A relative standard deviation is 2.4% ($n = 10$) for 200 ppm (v/v) of nitrogen dioxide. The sampling frequency is about 24 $h^{-1}$.*

### Introduction

Flow injection analysis (FIA) is based on the injection of a liquid sample into a moving unsegmented continuous carrier stream of a suitable liquid. The injected sample forms a zone, which is transported toward a detector. The hallmark of FIA is that dispersion is reproducible and controllable. Now FIA has evolved into a general technique for solution handling and data gathering, applicable to many areas of chemical research and technology [1]. FIA was mainly used in the analysis of liquid samples. The analysis of gaseous samples is attended by special difficulties [2]. Absorption by suitable solutions is usually used to collect and/or preconcentrate the analyte prior to the measurement. Only a few flow injection methods have been reported for the determination of gaseous samples. These methods can be assembled into these groups: (1) the gaseous sample is directly inserted into a flow injection system and reacts at a gas/liquid interface [2–5], or at a gas/solid interface [6]; (2) a dual phase gas diffusion/permeation technique which involved a liquid donor and gaseous acceptor [7–10] or the reverse [11–15] is employed by means of a separated membrane; and (3) a gas sensor is directly used as the detector in FIA [16, 17].

Nitrogen dioxide is a major air pollutant that plays a dominant role in acid deposition chemistry, as well as in the production of ozone and hydroxyl radicals [18]. A number of analytical methods has been developed suitable for emission control and the determination of low levels occurring in the atmosphere. The widely adopted instrumental chemical method for gaseous pollutants is coulometric analysis [19]. This involves measurement of the electrical current produced when strongly oxidizing or reducing pollutant gases react with potassium iodide or bromide solution in an electrochemical cell [20–22]. The gaseous sample is drawn through the detector containing a platinum cathode and a carbon anode with a galvanic potential difference between them. The current in the anode–cathode circuit is proportional to the amount of $NO_2$ entering the cell [21–24]. This type of galvanic detector was designed for continuous monitoring of nitrogen dioxide for a steady state measurement. The present paper describes a study on the features of the galvanic detector of nitrogen dioxide in flow injection analysis. The results of FIA are compared with the usual continuous flow monitoring. While the method of sample introduction changes from continuous flow monitoring (steady state) to FIA, the reproducible peak signals are obtained. The FIA method is simple, rapid and extending the measuring range of the galvanic detector. The effect of FIA variables, i.e. flow rates of sample and carrier, sample volume, delay time and calibration curve, is examined.

### Experimental

### *Gases*

A certified gas of nitrogen dioxide was obtained from the National Research Centre for Certified Materials (NRCCM, Beijing, China), and the certified value was 0.103% $NO_2/N_2$, mol/mol (No. 406850). The mixtures of nitrogen dioxide with air were also prepared in a 10-litre glass bottle connected to an air-pump and sampling coil of the injected valve in the sample enclosure, and were calibrated by means of the certified nitrogen dioxide. Air filtered by the scrubbers of silica gel and activated charcoal was used as the gaseous carrier. CO (500 ppm in $N_2$) was obtained from a cylinder (NRCCM). The gases $SO_2$, $H_2S$ and $CO_2$ were produced by chemical reactions of their respective salts with a solution of sulphuric acid, and were separately prepared in the 10-litre glass bottle containing air.

### *Apparatus*

The samples of nitrogen dioxide were detected by a $NO_2$ galvanic detector (The Third Analytical Instrument Factory of Tianjin, China) and a schematic diagram of the component is given in figure 1(a). The principle of the detector was the coulometric internal electrolysis [21–24]. The detector utilized a cyclic oxidation–reduction process and contained a platinum gauze cathode and carbon anode. The composition of the electrolyte consisted of 0.3 mol/l potassium iodide in a neutral

‡ Address for correspondence.

This paper was presented at the Seventh International Conference on Flow Analysis, Piracicaba, Brazil, 25–28 August 1997.

0142-0453/98 $12·00 © 1998 Taylor & Francis Ltd

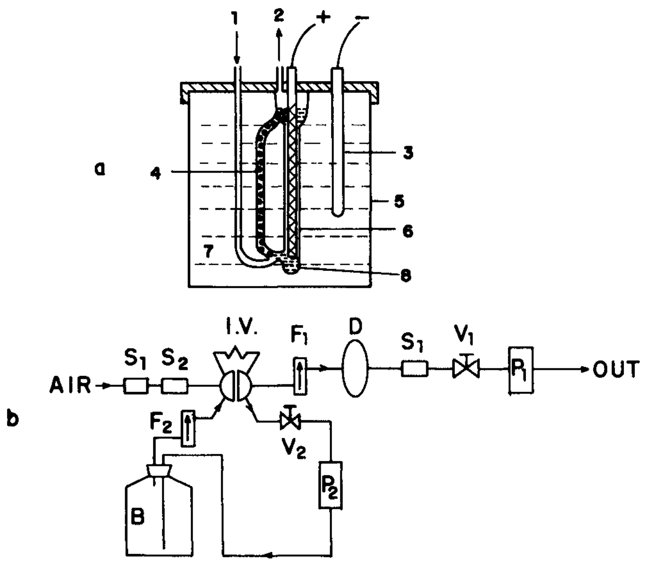

*Figure 1. (a) A side view of the galvanic detector of nitrogen dioxide. 1 = sample inlet; 2 = sample outlet; 3 = activated charcoal anode; 4 = gaseous bubble; 5 = plexiglas receptacle; 6 = platinum gauze cathode; 7 = neutral buffered electrolyte; 8 = small hole. (b) Scheme of system for the determination of $NO_2$ by flow injection. Flow direction is indicated by arrows. $S_1$ = silica gel scrubber; $S_2$ = activated charcoal scrubber; I.V. = six-port rotary injection valve; $F_1, F_2$ = flow meter; D = $NO_2$ galvanic detector; $V_1, V_2$ = needle valve; $P_1, P_2$ = air pump and B = 10-litre glass bottle.*

(pH = 7.0) phosphate buffer solution. The features of the detector claimed by the manufacturer are: measuring range of 0–4 mg/m$^3$ (about 2 ppm); detection limit 0.02 mg/m$^3$; response time (90% of final value) less than 4 min; precision better than 4%. The analytical system was composed of two electromagnetic air-pumps and a six-port rotary valve of stainless steel (SP-2305 for gas chromatography, the Analytical Instrument Factory of Beijing, China) supplied with 0.5, 1.0, 2.0 and 3.0 ml sampling coilings used to introduce the gaseous samples. The carrier and sample streams were aspirated by the pumps. Flow rates of sample and carrier were indicated by the two flowmeters (701 HB, range of 0.05–0.50 l/min, Beijing and LZB-3T, range of 0.1–1.0 l/min, Changzhou, China). The operational amplifier circuit was laboratory-made. The signal peak was displayed by a chart recorder (Dahua, Shanghai). All measurements were performed at room temperature, about 15 °C.

### *Manifold*

The scheme of the flow injection system is shown in figure 1(*b*). The clean air obtained by flowing the air through activated charcoal ($S_1$) and silica gel ($S_2$) filters was continuously aspirated as carrier through the galvanic detector (D). The gaseous mixture of nitrogen dioxide was circulated within the sample enclosure and the loop of the injection valve was filled with the sample by means of an air-pump (P2). The flow rates of carrier and sample were adjusted by the adjusting valves ($V_1, V_2$) to 0.25 and 1.0 l/min, respectively. Various volume samples of 0.5–3.0 ml were injected into the air carrier. The peak signals of the galvanic cell current were monitored as the potential drop across a precision resistor.

### Results and discussion

The galvanic detector of nitrogen dioxide was usually employed in the continuous flow monitoring, and the steady state signal was necessary. When the detector is used in a flow injection manifold, a typical peak graph of FIA is observed [25]. The printout of multiple injections of $NO_2$ sample is reproducible for a given concentration of nitrogen dioxide. The hallmark of a conventional FIA experiment is that dispersion is reproducible and controllable. By observing the peak shapes and heights it was clear that the sample zone reproducibly expanded or diluted in the flowing stream. Since identical physical and chemical conditions are consistently obtained in FIA, the steady state is not necessary. A six-port rotary sample valve and syringe were tested for the introduction of gaseous samples. For 200 ppm (v/v) $NO_2$, the relative standard deviations are 2.4% ($n = 10$) for the quantitative sampling valve and 14.3% ($n = 8$) for the syringe, respectively. The result shows that for the gaseous analysis the method of sample introduction has greatly influenced the reproducibility and that the six-port quantitative sample valve is better than the syringe. Similar results were also obtained for the determinations of sulphur dioxide by means of a gas sensor in FIA [17]. Therefore, the six-port rotary valve was used in all the experiments.

### *Effect of flow rates*

In continuous monitoring analysis, the steady state is necessary and the output current of the galvanic detector for $NO_2$ is proportional to the nitrogen dioxide concentration in the following equation [22–24] (derived from Faraday's law):

$$I/\mu A = 0.0669 \times f/(\text{ml/min}) \times c/(\text{ppm}) \quad (1)$$

where $I$ is the galvanic current in µA: $f$ is the sample flow rate in ml/min at 20 °C and 1013 mbar; and $c$ is the nitrogen dioxide concentration in ppm (v/v). In a certain range of flow rate, for a given concentration of nitrogen dioxide the output current is dependent on flow rate and linearly increases with flow rates [22].

When the detector is used in the flow injection mode, a graph of the signal as a function of flow rates, for sample and carrier, shows the different behaviours in comparison with that in common continuous flow monitoring mode (steady state, figure 2). At a constant sample flow rate, 1.0 l/min, the increase of the flow rate of the carrier results in a small initial signal increase at 0.20 l/min followed by a continuous linear decreasing (curve B). Simultaneously, a little increasing of the residual currents (curve C) is observed. In the range of carrier flow rate, from 0.20 to 0.45 l/min, the relationship of the response signal and the flow rate is given by the equation:

$$I/\mu A = (5.39 \pm 0.04) - (5.17 \pm 0.11) \times f/(\text{l/min}) \quad (n = 6) \quad r = 0.9991 \quad (2)$$

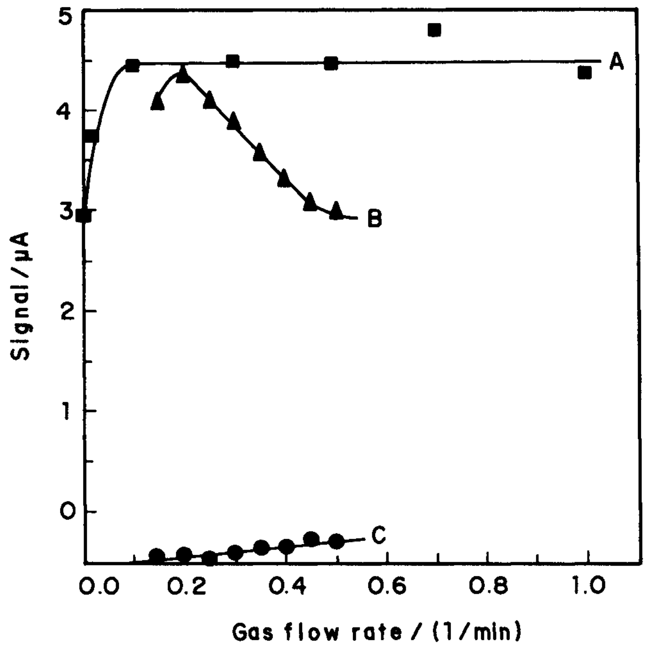

*Figure 2. Effect of flow rates of sample (A) and carrier (B and C) on the signals. 200 ppm $NO_2$, 0.5 ml sample volume. A: influence of sample flow rate, carrier at the flow rate of 0.25 l/min; B and C: effect of carrier on the responses of $NO_2$ and air, sample at the flow rate of 1.0 l/min.*

This is explained, at higher flow rates of the carrier, by the increasing of the dispersion of the sample zone in the tubes and the shorter reacting time in the detector. While the flow rate of the carrier is constant at 0.25 l/min, the analytical signal is essentially independent of the flow rate of the sample except at very low flow rates (curve A). This behaviour shows that, above certain sampling rates, it is not necessary to provide precise flow control of the sample. When the sample is maintained in the stationary mode or at lower flow rates (i.e. less than 0.10 l/min), the signal is smaller. This is perhaps caused by the absorption of nitrogen dioxide by the sample coil.

*Analytical frequency*

The effect of the interval $\Delta t$ of delay time on the signal reproducibility was examined (table 1). The first sample was injected into the carrier and the first signal peak ($i_0$) obtained. After the time interval $\Delta t$, the second sample was injected and obtained the second signal peak ($i$). When $\Delta t$ is longer than about 100 s, the relative difference of the two signals is smaller than 5%. Therefore, the minimum time interval between sample introductions must be about 2.5 min to obtain the relative difference between $i$ and $i_0$ less than 1.5%. Because the physical mixing and chemical conditions can be carried out reproducibly in FIA, the steady state signal is not necessary. In comparison with that of continuous flow monitoring, the analytical rate of FIA is increased. The sampling frequency is $24\,h^{-1}$.

*Sample volume*

The change of the injected sample volume is a powerful way to change dispersion. Increasing the volume of the injected sample results in higher peak height and sensitivity [1]. The effect of sample volume on the signal at various $NO_2$ concentrations is shown in figure 3. Below 1.0 ml of sample volume, the peak height for various concentrations of $NO_2$ is linearly proportional to the injected sample volumes; above 1.0 ml the signal increases gradually with the sample volume. The increase of sensitivity is limited by the rate of adsorption and reaction of nitrogen dioxide at the electrode. Therefore, further increasing sampling volume increases little sensitivity and causes a decreasing of the analytical frequency. The optimum sample volume is considered as 1.0 ml.

*Table 1. Effect of delay time interval $\Delta t$ on the reproducibility*.*

| $\Delta t$/s | 45 | 90 | 98 | 105 | 113 | 126 | 150 | 185 |
|---|---|---|---|---|---|---|---|---|
| $i/i_0$ | 1.249 | 1.069 | 1.048 | 1.035 | 1.036 | 1.032 | 1.015 | 1.003 |

* 200 ppm $NO_2$, 0.5 ml sample volume, carrier: 0.25 l/min, sample: 1.0 l/min. $i$ is the signal of second sample injected after the time interval $\Delta t$ of the first sample $i_0$.

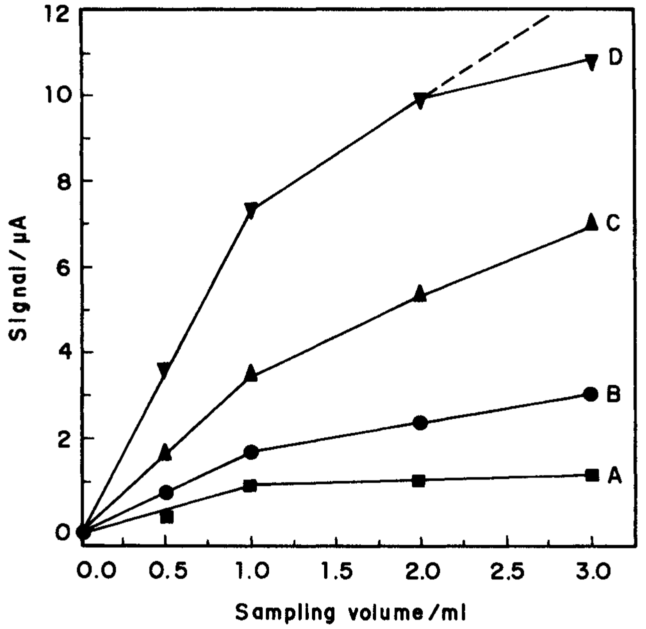

*Figure 3. Relationship of the signal and sample volume for various concentrations of $NO_2$. The concentrations of $NO_2$ for curves A, B, C and D are 20, 50, 100 and 200 ppm of $NO_2$, respectively. Carrier = 0.25 l/min, sample = 1.0 l/min.*

*Calibration curves*

The calibration plots for the determination of nitrogen dioxide, with various sample volumes under the optimum conditions, are shown in figure 4. The features of the determination of $NO_2$ are summarized in table 2. The measuring range of the galvanic detector was designed to monitor continuously $NO_2$ up to 2 ppm. The application of FIA extends the measuring range of nitrogen dioxide up to 500 ppm (v/v), with a precision, expressed as relative standard deviations of 2.4% for 200 ppm (v/v) [$n = 10$, sample volume 0.5 ml]. The detection limit is about 1 ppm of nitrogen dioxide. The relative sensitivity

*Table 2. Features of the calibration graphs obtained for the various sample volumes.*

| *Sample volume/ml* | *Range /ppm* | *Equation** | *Points of determination* | *Correlation coefficient* | *Detection limit/ppm* | *Relative sensitivity /[μA/(ppm·ml)]* |
|---|---|---|---|---|---|---|
| 0.5 | 0–500 | $I = -0.180 + 0.0188C_{NO_2}$ | 6 | 0.9996 | 4.2 | 0.0376 |
| 1.0 | 0–400 | $I = 0.243 + 0.0375C_{NO_2}$ | 8 | 0.9985 | 2.2 | 0.0375 |
| 2.0 | 0–200 | $I = -0.083 + 0.0506C_{NO_2}$ | 6 | 0.9985 | 1.6 | 0.0253 |
| 3.0 | 0–100 | $I = -0.354 + 0.0714C_{NO_2}$ | 5 | 0.9974 | 1.1 | 0.0238 |

* $I$ in μA and $C_{NO_2}$ in ppm (v/v).

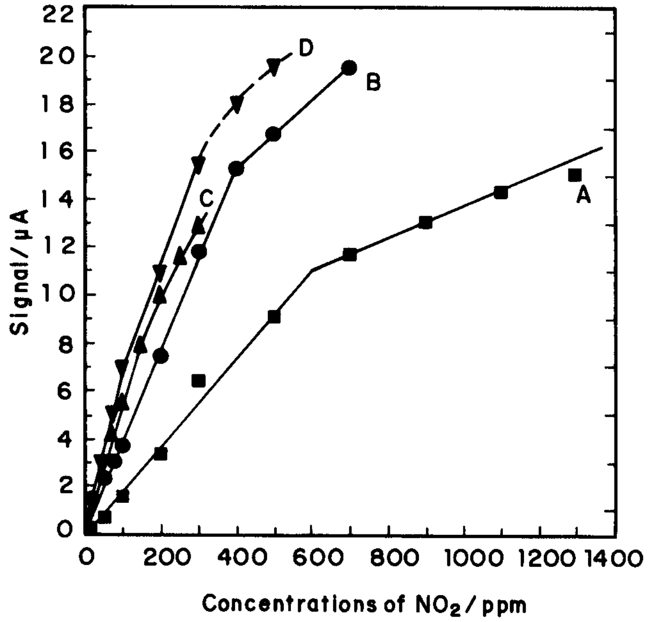

*Figure 4. Calibration curves with various sample volumes. A, B, C and D represent the sample volumes of 0.5, 1.0, 2.0 and 3.0 ml, respectively. Carrier = 0.25 l/min, sample = 1.0 l/min.*

of the galvanic detector in FIA depends on the sampling volume. The measuring range and sensitivity of the FI system can be easily varied by changing the injected sample volume.

### *Influence of other gases*

In the continuous monitoring of $NO_2$ a positive interference is given by $O_3$ and $Cl_2$, and a negative interference by $SO_2$, $H_2S$ and other sulphides [19]. The influence of CO, $CO_2$, $SO_2$ and $H_2S$ was tested in flow injection analysis, and the results are given in table 3. The existence of CO (500 ppm) and $CO_2$ (1800 ppm) does not interfere the determination of $NO_2$ (200 ppm). With the presence of 200 ppm of $SO_2$ or $H_2S$, the signals of $NO_2$ are completely covered, and a reproducible negative peak can be observed (figure 5). However, the peak shape with $SO_2$ or $H_2S$ is different from that of $NO_2$ as a tail appears. Because the suitable selective sample scrubbers have been designed by commercial manufacturers to remove interfering substances in continuous flow monitoring [19], the selectivity of FIA may be enhanced by the connection of a gas permeation device and a selective scrubber inserted into the acceptor [26].

*Table 3. The response signals of other gases.*

| *Gases/ppm (v/v)* | *Normalized signal* | *Relative error/%* |
|---|---|---|
| $NO_2$ (200) | 1.000 | |
| $NO_2$ (200) + $CO_2$ (1800) | 0.977 | −2.3 |
| $NO_2$ (200) + $SO_2$ (200) | −0.38 | −138 |
| $NO_2$ (200) + $H_2S$ (200) | −0.54 | −154 |
| $NO_2$ (200) + CO (500) | 1.016 | 1.6 |

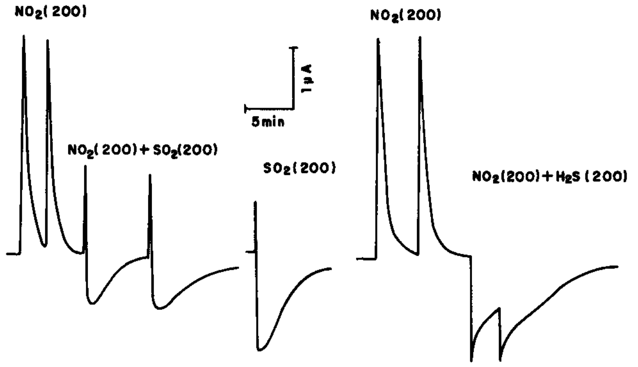

*Figure 5. Effect of $SO_2$ and $H_2S$ on the determination of $NO_2$ in FIA by the galvanic detector. The numbers represent the sample concentrations in ppm (v/v). Sample volume = 0.5 ml except for 200 ppm $SO_2$: sample volume 1.0 ml.*

### Conclusions

The galvanic detector of nitrogen dioxide is suitable for the flow injection analysis of nitrogen dioxide, extending the applications of the detector. The FI method speeds up the analytical process because identical physical and chemical conditions are consistently obtained, and the steady state is not required. The optimum sampling volume for the nitrogen dioxide galvanic cell in flow injection analysis (FIA) is 1.0 ml. In FIA the syringe is not suitable for the introduction of gaseous samples, while a quantitative rotary sampling valve gives good reproducibility. The measuring range and sensitivity of flow injection systems are easily adjusted by changing the sample volume.

### Acknowledgement

S. J. Liu wishes to thank Conselho Nacional de Desenvolvimento Científico e Tecnológico (CNPq), Brazil, for a grant (No. 150109/95-4).

### References

1. Ruzica, J. and Hansen, E. H., 1988, *Flow Injection Analysis*, 2nd edn. (New York: Wiley).

2. Cañete, F., Ríos, A., Luque de Castro, M. D. and Valcárcel, M., 1989, *Analytica Chimica Acta*, **224**, 127.

3. Ramasamy, S. M. and Mottola, H. A., 1982, *Analytical Chemistry*, **54**, 283.

4. Ríos, A., Luque de Castro, M. D., Valcárcel, M. and Mottola, H. A., 1987, *Analytical Chemistry*, **59**, 666.

5. Nyasulu, F. W. and Mottola, H. A., 1987, *Journal of Automatic Chemistry*, **9**, 46.

6. Ramasamy, S. M., Jabbar, S. M. A. and Mottola, H. A., 1980, *Analytical Chemistry*, **52**, 2062.

7. Canham, J. S. and Pacey, G. E., 1988, *Analytica Chimica Acta*, **214**, 385.

8. Canham, J. S. and Pacey, G. E., 1988, *Analytical Letters*, **21**, 1619.

9. Pacey, G. E., Straka, M. R. and Gord, J. R., 1986, *Analytical Chemistry*, **58**, 502.

10. Aoki, T. and Wakabayashi, M., 1995, *Analytica Chimica Acta*, **308**, 308.

11. Smith, K. J. and Pacey, G. E., 1993, *Talanta*, **40**, 1961.

12. Kubán, V. and Dasgupta, P. K., 1992, *Analytical Chemistry*, **64**, 1106.

13. Liu, S. and Dasgupta, P. K., 1995, *Analytica Chimica Acta*, **308**, 281.

14. Aldstadt, J. H., Olson, D. C. and Martin, A. F., 1997, *Analytica Chimica Acta*, **338**, 215.

15. Van Nugteren-Osinga, I. C., Bos, M. and Van der Linden, W. E., 1989, *Analytica Chimica Acta*, **226**, 171.

16. Mills, A. and Lawrence, C., 1984, *Analyst*, **109**, 1549.

17. Liu, S. J., Shen, H. X. and Feng, J. X., 1996, *Acta Chimica Sinica*, **54**, 813.

18. Schepers, D., Schulze, G. and Frenzel, W., 1985, *Analytica Chimica Acta*, **308**, 109.

19. Perry, R. and Young, R. J. (Eds), 1977, *Handbook of Air Pollution Analysis* (London: Chapman and Hall), 232–234.

20. Hersch, P. and Deuringer, R., 1963, *Analytical Chemistry*, **35**, 897.

21. Hersch, P and Deuringer, R., 1967, US Patent 3,314,863.

22. Allen, J. D., 1974, *Analyst*, **99**, 765.

23. Department of Chemistry, Beijing University, 1976, *Huaxue Tongbao*, **2**, 22.

24. Dong, L., Liang, J. M. and Gao, X. X., 1979, *Fenxi Yiqi*, **4**, 14.

25. Lui, S. J., Shen, H. X., Ding, X. C. and Feng, J. X., 1995, *Chinese Chemical Letters*, **6**, 975.

26. Liu, S. J., Shen, H. X., Feng, J. X. and Tubino, M., 1997, *Fresenius Journal Analytical Chemistry*, **357**, 1045.

